# To the Editors of the Medical and Physical Journal

**Published:** 1804-10-01

**Authors:** J. J. Smith

**Affiliations:** Plymouth-Dock


### *To the Editors of the Medical and Physical Journal.*

GENTLEMEN,

I Have taken the liberty of sending you a statement of the following case of wounded brachial artery, the most essential point in the treatment of which, I learnt from a communication in the Medical Journal, in the month of July, 1802.

I am, &c.

*Plymouth-Dock.* J. J. SMITH.

---

CASE.—Edward Kelly, on the 8th of December, 1802, wounded himself with a razor in the arm near the usual place of venæsection, carrying the instrument to such a depth, as partially to divide the brachial artery: The hæmorrhage had almost proved fatal when I first visited him, and I had immediate recourse to the effects of pressure, which in the present languid state of my patient succeeded in arresting the bleeding.

On the eighth day afterward, a slough took place from the wound, and a profuse bleeding succeeded, for which the tourniquet was applied; and as nothing but an operation afforded the hope of saving the life of my patient, I immediately performed one, for the purpose of tying the artery. The arm was much distended with extravasated blood, it was cold and livid, and vesications had already formed near the wound, which had discharged a bloody ichor, and shewed a state approaching to gangrene. I made a longitudinal incision of four inches across the wound inflicted by the razor, which gave me room to proceed in the after steps of the operation, and afforded an opportunity of removing the coagulated blood with which the arm was loaded.

The fascia being laid bare, and then cautiously divided, exposed the ends of the wounded artery in such a manner that the vessel could be readily secured. A ligature was passed around the upper part of the vessel near the orifice, and this being tied in a knot, and the end of it being armed with a needle, the needle was thrust through the artery below the circular ligature, and the thread was tied into the knot previously made upon the vessel.

The same was then effected in the other end of the

 artery, artery, which was now an inch below the first. The wound was dressed superficially with the ligatures hanging from it. On the eleventh day the ligatures came away, and the wound was healed in two months, being prevented by a bad constitution from uniting sooner; the motion of the elbow is perfect, and the arm as useful as before, if allowance is made for its being slightly weaker under any great exertion. The security which is obtained by passing the ligature through the artery, nearer the orifice than where the thread is circularly applied, induced me to adopt that practice in the case which I have related, and some such security seems to be necessary, from the cases of hæmorrhage which have happened after the operation for the aneurism, and which have been also known to occur after amputations, which have been performed so near to the body, as to expose the ligature to great impetus of blood from the action of the heart; for I lately witnessed an instance where the arm was amputated near the shoulder joint, and the brachial artery considered as secured; when, upon loosening the tourniquet, a violent bleeding succeeded from the ligature being forced off the extremity of the vessel, but which fortunately happening before the surgeon quitted the room, the life of the patient did not become a sacrifice.

---

